# The Incidence of Urgent Tube Shunt Surgery for Diabetic Neovascular Glaucoma at a Tertiary Academic Medical Center

**DOI:** 10.1177/11795514231203865

**Published:** 2023-10-27

**Authors:** Megan Corinne LaRocca, Andrew K Smith, Don S Minckler, Ken Y Lin

**Affiliations:** 1 University of California, Irvine School of Medicine, Irvine, CA, USA; 2 Gavin Herbert Eye Institute, Department of Ophthalmology, UC Irvine, Irvine, CA, USA; 3 Department of Biomedical Engineering, UC Irvine, Irvine, CA, USA

**Keywords:** Ahmed glaucoma implant, glaucoma emergency, glaucoma drainage implant, aqueous shunts, diabetic retinopathy

## Abstract

**Background:**

Diabetic neovascular glaucoma is a secondary glaucoma that may require immediate correction of elevated intraocular pressure to control pain and protect the optic nerve. While there is a seasonal trend to glucose levels, it is unknown if a seasonal trend exists for diabetic neovascular glaucoma.

**Objective:**

This study evaluates the incidence of urgent glaucoma tube shunt implantation in diabetic neovascular glaucoma in a tertiary academic referral center in Southern California.

**Methods:**

Electronic medical records were queried for urgent glaucoma tube shunt surgery from 2014 to 2021. The number of cases were separated by month of occurrence, and average hemoglobin A1c values were calculated per month. Data were analyzed via ANOVA tests and one-tailed t-tests.

**Results:**

A total of 127 cases were identified. The months of March and April contained the most cases averaging 3 and 2.75 cases, respectively. April had statistically significant higher case numbers than that of other months (*P* = .041). ANOVA tests excluding April showed no statistically significant difference between the remaining months (*P* = .901). Average hemoglobin A1c values were highest in the months of April and March at 9.8 and 9.6%, respectively.

**Conclusion:**

Emergency glaucoma tube shunt surgery for diabetic neovascular glaucoma occurs most frequently in April. This observation may provide insight into disease prevention through diabetes management and help improve surgical operations such that staffing and resources are allocated accordingly.

## Introduction

Neovascular glaucoma (NVG) is a secondary glaucoma caused by obstruction of aqueous humor outflow at the anterior chamber angle by peripheral anterior synechiae (PAS) provoked by retinal ischemia.^1^ During neovascular remodeling, profibrotic markers upregulate smooth muscle alpha-actin prompting the differentiation of fibroblasts into myofibroblasts.^2,3^ This newly developed fibrovascular membrane maintains contractile forces that reduce wound size and lead to potential anatomical complications such as angle closure.^4^ Patients often present in the acute setting with elevated intraocular pressure (IOP) and corneal edema with severe ocular pain. While paracentesis with maximal medical therapy may temporize the emergency, NVG often requires prompt surgical management. Chronic management requires decreasing the vascular endothelial growth factor (VEGF) burden generated by ischemic retina as well as controlling the IOP.

The University of California, Irvine (UCI) Medical Center is the only level-one adult trauma center in Orange County (OC), and most adult ophthalmic urgent surgeries in the county are referred there. The greater OC area consists of 34 cities and a diverse population with large White, Hispanic, and Asian representations (Table 1) with a total population of over 3 million people.^5,6^ OC’s gender and age proportions are about 50.7% female and 15.3% above the age of 65.^6^ At UCI Medical Center, urgent glaucoma surgery is performed when a patient’s IOP is refractory to medical therapy in the acute setting. When a patient presents with ocular hypertension from NVG, maximal medical therapy including a systemic carbonic anhydrase inhibitor is administered. If the patient’s IOP is not responsive as indicated by an IOP greater than 30 mmHg by 90 minutes after maximal medical therapy is administered, an urgent glaucoma tube shunt implantation is arranged. This procedure frequently takes place in the main operating facility shared by all other surgical services. An intravitreal injection of bevacizumab is given in addition to anterior chamber paracentesis at the slit lamp or at the conclusion of the tube-shunt surgery intraoperatively. All emergency NVG tube shunt cases at UCI utilize either an FP7 Ahmed or 350 Baerveldt device at the discretion of the surgeon.

**Table 1.** Demographic composition in Orange County.^5^

Demographics	Percent
White alone, percent	71.1
Black or African American alone, percent	2.1
American Indian and Alaska Native alone, percent	1.0
Asian alone, percent	21.7
Native Hawaiian and Other Pacific Islander alone, percent	0.4
Two or More Races, percent	3.6
Hispanic or Latino, percent	34.0
White alone, not Hispanic or Latino, percent	39.8

The population contains a total of 3 175 692 people. Those of the race “White alone” make up the majority of the population. Data was reported on July 1, 2019.

To date, no seasonal trend for NVG has been documented in published reports. In several studies, there was a strong relationship between fasting glucose levels and seasonality.^7,8^ Specifically, winter and spring were associated with higher glucose levels than that in summer and autumn.^7^ Hemoglobin A1c levels were also noted to be higher in the winter and spring months peaking from February to early April and falling from August to October.^9^ Although many disease processes can result in NVG, one of the leading causes of NVG is proliferative diabetic retinopathy (PDR) (33-48.7%).^10,11^ It is unknown if higher glucose levels in the winter and spring may lead to greater incidents of NVG. Identifying seasonal trends of NVG can be advantageous in terms of implementing preventative medicine and resource planning. If surgical cases for NVG showed temporal or seasonal clustering, appropriate call-coverage, resources, and patient education can be planned and allocated accordingly.

To this end, this paper seeks to analyze the seasonal incidence of urgent glaucoma tube shunt surgery to treat diabetic NVG at UCI Medical Center through a retrospective analysis from 2014 to 2021.

## Materials and Methods

### Data collection

Data regarding NVG tube shunt surgeries at UCI Medical Center was collected from Medflow Electronic Medical Record (Charlotte, North Carolina) and the Gavin Herbert Eye Institute surgery scheduler case logs from 2014 to 2017. UCI Medical Center switched its electronic medical record system to EPIC (Verona, Wisconsin) beginning in November 2017. A customized query of EPIC was performed using the following parameters: encounters with CPT Code 66180 (glaucoma drainage device with placement of a scleral patch graft), ICD10 codes H40.50X0-4, H40.51X0-4, H40.52X0-4, H40.53X0-4 (neovascular glaucoma), location of surgery at the main operating room, and priority of surgery classified as “emergent or “urgent,” having “diabetes” in the medical problem list were searched. This ensured subjects included in the study were diabetic patients with NVG receiving urgent aqueous tube shunt surgery at UCI Medical Center from 2014 to 2021. The sample size included all patients receiving surgery who sustained an IOP greater than 30 mmHg in the affected eye after medical therapy. Subjects excluded from this study included those who refused surgery, responded within 90 minutes to maximal medical therapy, or presented without light perception in the affected eye. Of the cases identified, the patients’ race and hemoglobin A1c values were also collected.

### Statistical analysis

The identified cases and the corresponding hemoglobin A1c values were separated by which month the surgery occurred. ANOVA tests were conducted for every month and again for every month excluding April to evaluate for a statistically significant difference between the number of cases per month. The power of the ANOVA for all months was calculated using a noncentral F distribution. Two-tailed, unequal variances *t*-tests were then performed to evaluate the differences between NVG surgeries in March, April, and March and April versus the remaining months of the year. The average values of hemoglobin A1c were calculated by month. Statistical analysis was performed via Prism 9 (GraphPad Software, San Diego, CA). Surgical case values were expressed as average ±standard error of the mean (SEM).

## Results

**Figure 1.**
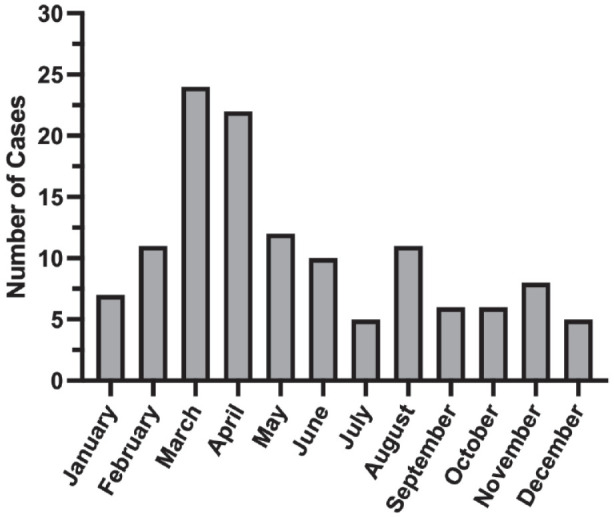
The total number of diabetic neovascular glaucoma tube shunt surgeries by month from 2014 to 2021 (n = 127). March and April had the greatest number of cases totaling 24 and 22 cases, respectively. The total number of cases performed in April was significantly greater than that of the remaining months (*P* = .021).

**Figure 2.**
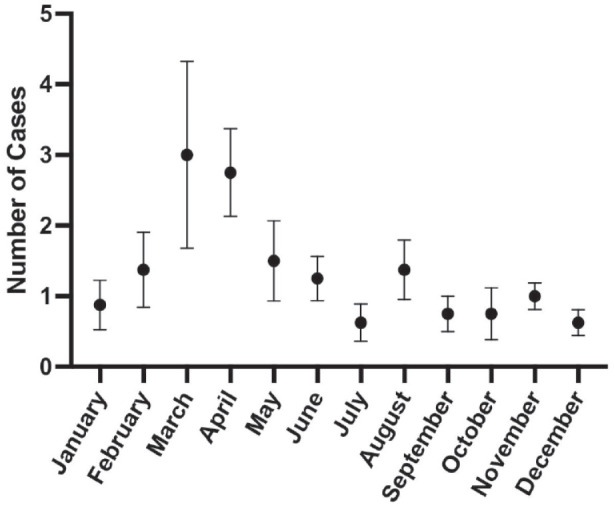
The average number of diabetic neovascular glaucoma tube shunt surgeries by month from 2014 to 2021. Error bars represent SEM. April and March had the highest average of tube shunt surgeries at 3 and 2.75 cases, respectively, per month.

One hundred twenty-seven cases met the inclusion criteria. The number of cases in March and April were greater than that of the remaining months of the year totaling 24 and 22 cases, respectively (Figure 1). On average, 1.32 surgeries were performed each month during the eight-year period. Excluding March and April, an average of 1.01 urgent tubes were performed per month. In contrast, the average number of cases performed in March and April were 3 and 2.75 respectively with the mode number of cases being 2 in each month (Figure 2). The next highest month was May in which an average of 1.5 cases were performed. Of the 127 cases, the majority of patients (79%) were Hispanic or Latino (Table 2).

**Table 2.** Demographic composition of the diabetic patients undergoing tube shunt surgery at UCI.

Demographics of patients	Percent
Hispanic or Latino	79.0
White alone, not Hispanic or Latino	5.0
Black or African American alone	5.0
Asian alone	3.3
Other, unknown, unspecified	7.7

The majority of patients were those of the race “Hispanic or Latino.”

ANOVA analysis of every month indicated populations were not equal suggesting that at least 1 month was statistically different from the rest (*P* = .025). The power of the ANOVA study analyzing all months was 89.8%. The number of cases performed in April (*P* = .041) as well as March and April (*P* = .019) were statistically significantly greater when compared to those of the remaining months (April, *P* = .041; March and April, *P* = .019). Although March maintained the highest number of cases, the value was not statistically significant (*P* = .210). The number of cases performed in July and December were significantly less than the cases performed in the remaining months (July *P* = .030; December, *P* = .0064). Lastly, ANOVA analysis of every month excluding April as well as March and April showed no statistically significant differences between the remaining months (April, *P* = .901; March and April, *P* = .375).

**Figure 3.**
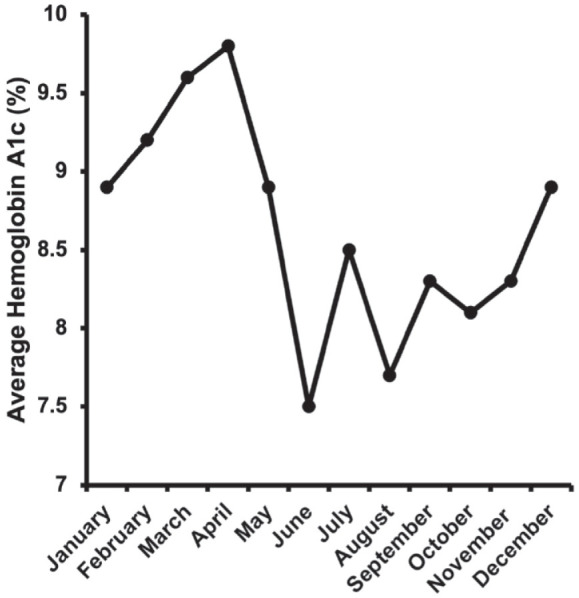
The average hemoglobin A1c values by month from 2014 to 2021. April and March had the highest averages at 9.8 and 9.6%, respectively. August and June had the lowest averages at 7.7 and 7.8%, respectively.

The average hemoglobin A1c values by month from 2014 to 2021 are depicted in Figure 3. The highest hemoglobin A1c peaked during the months of April and March at 9.8 and 9.6%, respectively. June and August retained the lowest hemoglobin A1c values at 7.8 and 7.7%, respectively.

## Discussion

Our study reveals a statistically significant increase in urgent after-hour glaucoma tube shunt cases for NVG at a tertiary referral academic center during the months of April as well as March and April combined over an 8-year period. The total number of cases in March and April greatly exceeds the total cases in the remaining months of the year (Figure 1). The high power of the study further validated the study’s significance. Urgent tube surgery was performed on average once per month (1.01) when March and April are excluded. Conversely, in March and April, the average cases increased to 3 and 2.75 times per months (Figure 2). The mode for March and April was 2 cases while the other months’ mode was 1 case excluding July and October where the mode was 0 cases. The lowest number of cases was performed in July and December with an average of 0.625 cases per month. This may be due to decreased medical evaluations during the vacation and holiday seasons. This analysis indicates a significant rise in the number of urgent glaucoma tube shunt surgery performed for NVG in April and March and April combined.

Glucose levels and hemoglobin A1c values have been shown to worsen in the winter and spring.^7-9^ In our study, hemoglobin A1c values mimicked this trend, peaking generally in winter and spring months and falling in the summer months. As such, our study’s observations may be a sequela of uncontrolled diabetes during these periods. Elevation of hemoglobin A1c peaking in similar months to tube shunt surgeries (ie, March and April) suggests that higher incidence of NVG may be a consequence of chronically elevated blood glucose and long-standing neglect of glucose control.^9^ Increasing efforts to monitor patients’ blood glucose levels from a primary care standpoint during the winter and spring could lead to a decrease in NVG cases. Increasing eye exams for at-risk patients during these times also has the potential to detect neovascularization early and further prevent NVG. Early detection and management of NVG is pivotal to minimize complications such as vision loss and angle closure by neovascularization.^12^ This is especially important for at-risk populations, such as the Hispanic community in this particular study. Operating room personnel, residents, fellows, and attending staffing capacity can similarly be optimized in preparation for the rise in tube shunt cases in April and the fall in cases in July and December. This can include ensuring staff availability, coordinating proper call-coverage, and refreshing surgical knowledge and skills. It is important to note that tube shunt cases may continue to increase in number as studies are reinforcing a shift towards performing tube shunt surgeries over trabeculectomies.^13^ In addition, the prevalence of diabetes is predicted to rise from 537 million adults to 630 million adults in 2030.^14^ This would likely further magnify the number of NVG cases and subsequent tube-shunt cases performed in April. Such endeavors to optimize ocular surveillance and surgical resources could reduce NVG incidence and improve management of NVG.

### Study limitations

This study is not without limitations, including its retrospective nature. The total population collected was also used as the final sample size. It should be noted that not all cases of NVG require urgent tube shunt surgery and that urgent tube shunt surgery is not a surrogate marker for the true number of NVG cases seen throughout the area. In addition, PDR is not the only cause for NVG; ocular ischemic syndrome (OIS) and retinal vein occlusions (RVOs) can also lead to NVG. We recognized that OIS and RVOs can theoretically cause NVG in a diabetic patient. However, because PDR is the leading cause of NVG (33-48.7%) and we filtered for diabetic patients in our search, we concluded NVG due to PDR was the most likely etiology.^10,11^ The analysis also does not include patients who refused surgery or who do not undergo surgery urgently for other reasons such as a presentation of no light perception vision, but in our experience, this was extremely rare.

It is also important to note the year 2020 deviated from the observed trend. There were no cases of tube shunt surgeries performed in both March and April of 2020, which was unprecedented in the study period. We believe this may be attributed to the fact March 2020 was the beginning of the peak of the first wave of COVID-19 cases, and a state-wide stay-home order was issued March 19, 2020. This might have discouraged patients from seeking help.

## Conclusion

In conclusion, March and April harbored the most urgent glaucoma tube shunt cases performed for the treatment of diabetic NVG over an eight-year period. The number of cases performed in April were significantly greater than that of the remaining months. The reason for the rise of cases in these months may be related to uncontrolled blood glucose levels in the winter and spring months as correlated with peak hemoglobin A1c values in March and April. This information may help optimize resource allocation and patient monitoring during the high-risk months.

## Declarations

**Ethics approval and consent to participate:** The study protocol was approved by UC Irvine Biomedical IRB Committee B (approval number 20195254).

**Consent for publication:** Not applicable.

**Availability of data and materials:** Not applicable.
